# Quality of Life and the Experience of Living with Early-Stage Alzheimer’s Disease

**DOI:** 10.3233/JAD-220696

**Published:** 2022-11-08

**Authors:** Alberto Villarejo-Galende, Elena García-Arcelay, Gerard Piñol-Ripoll, Antonio del Olmo-Rodríguez, Félix Viñuela, Mercè Boada, Emilio Franco-Macías, Almudena Ibañez de la Peña, Mario Riverol, Albert Puig-Pijoan, Pedro Abizanda-Soler, Rafael Arroyo, Miquel Baquero-Toledo, Inmaculada Feria-Vilar, Mircea Balasa, Ángel Berbel, Eloy Rodríguez-Rodríguez, Alba Vieira-Campos, Guillermo García-Ribas, Silvia Rodrigo-Herrero, Ángeles Terrancle, Daniel Prefasi, Alberto Lleó, Jorge Maurino

**Affiliations:** a Department of Neurology, Hospital Universitario 12 de Octubre, Instituto de Investigación Hospital 12 de Octubre (I+12), Madrid, Spain; b Networking Research Center on Neurodegenerative Diseases (CIBERNED), Instituto de Salud Carlos III, Madrid, Spain; c Medical Department, Roche Farma, Madrid, Spain; d Cognitive Disorders Unit, Hospital Universitari Santa Maria de Lleida, Institut de Recerca Biomédica de Lleida (IRBLLeida), Lleida, Spain; e Department of Neurology, Hospital Universitario Dr. Peset, Valencia, Spain; f Instituto Neurológico Andaluz, Hospital Victoria Eugenia, Unidad Deterioro Cognitivo, Hospital Universitario Virgen Macarena, Sevilla, Spain; g Ace Alzheimer Center Barcelona, Universitat Internacional de Catalunya, Barcelona, Spain; h Dementia Unit, Department of Neurology, Hospital Universitario Virgen del Rocío, Instituto de Biomedicina de Sevilla (IBiS), Sevilla, Spain; i Centro de Investigación de Parkinson, Policlínica Guipúzcoa, San Sebastián, Spain; j Department of Neurology, Clínica Universidad de Navarra, Universidad de Navarra, Pamplona, Spain; k Cognitive Impairment and Movement Disorders Unit, Department of Neurology, Hospital del Mar, Hospital Del Mar Medical Research Institute (IMIM), Barcelona, Spain; l Department of Geriatrics, Complejo Hospitalario Universitario de Albacete, Albacete, Spain; m Department of Neurology, Hospital Universitario Quirónsalud, Madrid, Spain; n Grup d’Investigació en Malaltia d’Alzheimer, Department of Neurology, Hospital Universitari i Politècnic La Fe, Institut d’Investigació Sanitaria La Fe, Valencia, Spain; o Department of Neurology, Complejo Hospitalario Universitario de Albacete, Albacete, Spain; p Alzheimer’s Disease and other Cognitive Disorders Unit, Hospital Clínic, Institut d’Investigacions Biomèdiques August Pi i Sunyer (IDIBAPS), Barcelona, Spain; q Department of Neurology, Hospital Central de la Cruz Roja, Madrid, Spain; r Department of Neurology, Hospital Universitario de Marqués de Valdecilla, Instituto de Investigación Sanitaria Valdecilla (IDIVAL), Universidad de Cantabria, Santander, Spain; s Department of Neurology, Hospital Universitario de La Princesa, Madrid, Spain

**Keywords:** Alzheimer’s disease, amyloid, biomarkers, cerebrospinal fluid, magnetic resonance imaging, tau proteins, white matter hyperintensities, white matter lesions

## Abstract

**Background:**

There is a need to better understand the experience of patients living with Alzheimer's disease (AD) in the early stages.

**Objective:**

The aim of the study was to evaluate the perception of quality of life in patients with early-stage AD.

**Methods:**

A multicenter, non-interventional study was conducted including patients of 50–90 years of age with prodromal or mild AD, a Mini-Mental State Examination (MMSE) score ≥22, and a Clinical Dementia Rating-Global score (CDR-GS) of 0.5.–1.0. The Quality of Life in Alzheimer ’s Disease (QoL-AD) questionnaire was used to assess health-related quality of life. A battery of self-report instruments was used to evaluate different psychological and behavioral domains. Associations between the QoL-AD and other outcome measures were analyzed using Spearman’s rank correlations.

**Results:**

A total of 149 patients were included. Mean age (SD) was 72.3 (7.0) years and mean disease duration was 1.4 (1.8) years. Mean MMSE score was 24.6 (2.1). The mean QoL-AD score was 37.9 (4.5). Eighty-three percent (n = 124) of patients had moderate-to-severe hopelessness, 22.1% (n = 33) had depressive symptoms, and 36.9% (n = 55) felt stigmatized. The quality of life showed a significant positive correlation with self-efficacy and negative correlations with depression, emotional and practical consequences, stigma, and hopelessness.

**Conclusion:**

Stigma, depressive symptoms, and hopelessness are frequent scenarios in AD negatively impacting quality of life, even in a population with short disease duration and minimal cognitive impairment.

## INTRODUCTION

Alzheimer’s disease (AD) is currently one of the most important health problems due to an estimated prevalence of prodromal and AD dementia of 69 and 32 million people, respectively [1]. Despite different efforts to reframe the management of AD based on a patient-centered approach, patients are still given a passive role [2– 4]. Different drug development programs targeting amyloid and tau proteins in patients with AD are underway in recent years [5]. However, the measures traditionally included in these clinical trials are not sufficient to assess the full range of disease severity from the perspective of patients and their caregivers [2, 3, 6].

Subjective perception of life satisfaction and quality of life are indicators of the ability to live well in patients with chronic diseases [3]. Most patients with AD want to be informed of the diagnosis in order to plan their long-term care, to participate in medical decisions and clinical trials, and to maintain meaningful social relationships within their community [2, 7]. Quality of life is the priority for both patients with mild cognitive impairment and their partners, followed by maintaining their memory, mental status, and autonomy [6]. Optimism, self-esteem, and self-efficacy were associated with increased coping ability in patients with dementia [9].

Identifying the most meaningful outcomes for patients and their caregivers is essential to incorporate the voice of AD patients according to their beliefs and preferences [4, 7, 10]. The aim of this study was to assess the perception of quality of life in patients with early-stage AD using a battery of different patient-reported measurements.

## METHODS

We conducted a non-interventional, cross-sectional study at 21 hospital-based memory clinics in Spain. Patients between 50– 90 years old with a diagnosis of prodromal or mild AD (National Institute on Aging/Alzheimer’s Association criteria) [11], a Clinical Dementia Rating-Global score (CDR-GS) of 0.5 to 1.0 [12], and a Mini-mental State Examination (MMSE) score ≥22 [13] were included. The Research Ethics Board of Hospital de la Santa Creu i Sant Pau (Barcelona, Spain) approved the study design. Written informed consent was obtained from all participants.

### Outcome measures

The Quality of Life in Alzheimer’s Disease (QoL-AD) questionnaire [14] was used to assess health-related quality of life. Total score ranges from 13 to 52 with higher points indicating better quality of life. In addition, a battery of different self-report instruments was used to evaluate functioning (Functional Activities Questionnaire, FAQ) [15], mood (Beck Depression Inventory-Fast Screen, BDI-FS) [16], hopelessness (Beck Hopelessness Scale, BHS) [17], perception of stigma (Stigma Scale for Chronic Illness, SSCI-8) [18], understanding of the illness and its consequences (Representations and Adjustment to Dementia Index, RADIX) [19], the ability to overcome obstacles and setbacks or self-efficacy (General Self-Efficacy Scale, GSES) [20], and life satisfaction (Satisfaction With Life Scale, SWLS) [21]. The characteristics and scoring of each of these instruments have been published elsewhere [22].

### Statistical analysis

Demographic and clinical characteristics were summarized using descriptive statistics. Associations between the QoL-AD and other outcome measures were analyzed using Spearman’s rank correlations. Statistical significance was set at *p* < 0.05. The analysis was performed using IBM SPSS Statistics software version 22.0 (IBM Corp., Armonk, NY, USA).

## RESULTS

A total of 149 patients were included. Mean (SD) age was 72.3 (7.0) years and mean disease duration was 1.4 (1.8) years. Almost 90% of patients had a CDR-GS score of 0.5 with a mean MMSE score of 24.6 (2.1). Most participants were retired (79.2%) and independent for activities of daily living (72.5% with a FAQ score <9). Table 1 shows main sociodemographic and clinical characteristics of participants.

**Table 1:** Sociodemographic and clinical characteristics

	N = 149
Age, mean (SD), y	72.3 (7.0)
Female, *n* (%)	75 (50.3)
Marital status, married, *n* (%)	125 (83.9)
Working status, *n* (%)
Retired	118 (79.2)
Housewife	13 (8.7)
Partial or full-time employed	7 (4.7)
Education, mean (SD), y	13.1 (10.0)
Cardiovascular risk factors, *n* (%)	107 (71.8)
Hyperlipidemia, %	70.1
Arterial hypertension, %	59.8
Disease duration, mean (SD), y	1.4 (1.8)
MMSE score, mean (SD)	24.6 (2.1)
MMSE score distribution, *n* (%)
22– 24	79 (53.0)
≥25	70 (47.0)
ADAS-Cog13 score, mean (SD)	24.4 (5.2)
CDR-GS score, *n* (%)
0.5	130 (87.2)
1	19 (12.7)
FAQ score, mean (SD)	6.0 (6.2)
FAQ score <9^a^, *n* (%)	108 (72.5)
Use of anticholinesterase inhibitors, *n* (%)	86 (57.7)

ADAS-Cog13, Alzheimer's Disease Assessment Scale-Cognitive; CDR-GS, Clinical Dementia Rating-Global Score; FAQ, Functional Activities Questionnaire; MMSE, Mini-Mental State Examination; SD, standard deviation. ^a^A score <9 indicates independence for activities of daily living.

**Fig. 1:**
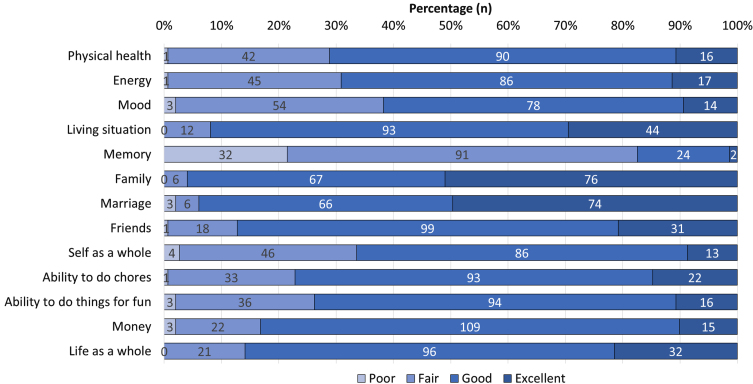
Quality of Life in Alzheimer’s Disease Scale domains.

Mean QoL-AD score was 37.9 (4.5) (Table 1), and memory was the domain with the highest negative impact, followed by mood, self as a whole, and energy (Fig. 1). Depressive symptoms were found in 22.1% (*n* = 33) of patients and moderate-to-severe feelings of hopelessness in 83.2% (*n* = 124) (Table 2). “I can’t imagine what my life would be in 10 years” and “Things just don’t work out the way I want them to” were the most frequent feelings of hopelessness (64.2% and 56.7%, respectively). The prevalence of stigma was 36.9% (*n* = 55) with low-to-moderate severity (Table 3). Similarly, participants reported their beliefs about AD and its emotional and practical consequences (Table 4). “People treat me differently”, “I do not go out as much as I used to”, and “I get very angry about what it is happening to me” were the most common self-reported consequences.

**Table 2:** Outcome measures

	N = 149
QoL-AD score^1^, mean (SD)	37.9 (4.5)
SWLS score, mean (SD)	27.5 (5.7)
SWLS score ≥25^a^, *n* (%)	120 (80.5)
BDI-FS score, mean (SD)	2.1 (2.2)
BDI-FS score ≥4^b^, *n* (%)	33 (22.1)
BHS score, mean (SD)	10.7 (2.4)
BHS score ≥9^c^, *n* (%)	124 (83.2)
SSCI-8 score, mean (SD)	2.1 (2.2)
SSCI-8 score ≥8^d^, *n* (%)	55 (36.9)
Practical consequences RADIX score^2^, mean (SD)	1.8 (0.6)
Emotional consequences RADIX score^2^, mean (SD)	2.2 (0.8)
GSES score^3^, mean (SD)	30.0 (6.3)

BDI-FS, Beck Depression Inventory-Fast Screen; BHS, Beck Hopelessness Scale; GSES, General Self-Efficacy Scale; QoL-AD, Quality of Life in Alzheimer’s Disease; RADIX, Representations and Adjustment to Dementia Index; SD, standard deviation; SSCI8, Stigma Scale for Chronic Illness; SWLS, Satisfaction With Life Scale. ^a^A score ≥25 indicates that respondents are satisfied or extremely satisfied with life; ^b^a score ≥4 indicates the presence of depressive symptoms; ^c^a score ≥9 indicates moderate-to-severe hopelessness; ^d^a score >8 indicates the presence of stigmatization. ^1^Total score ranges from 13 to 52 with higher points indicating better quality of life; ^2^total score ranges from 1 to 4 (practical) and 1 to 5 (emotional) with higher scores indicating greater negative consequences; ^3^total score ranges from 10 to 40 with higher scores indicating higher levels of optimistic self-belief.

**Table 3:** Perception of stigma (N = 149)

	Frequency, *n* (%)		
SSCI-8 items	Never/Rarely	Sometimes	Often/Always
Because of my illness, some people seemed	145 (97.3)	4 (2.7)	0 (0)
uncomfortable with me
Because of my illness, some people avoided me	145 (97.3)	4 (2.7)	0 (0)
Because of my illness, I felt left out of things	134 (89.9)	15 (10.1)	0 (0)
Because of my illness, people were unkind to me	146 (98.0)	3 (2.0)	0 (0)
Because of my illness, people avoided looking at me	149 (100)	0 (0)	0 (0)
I felt embarrassed about my illness	138 (92.6)	9 (6.0)	2 (1.3)
I felt embarrassed because of my physical	144 (96.6)	3 (2.0)	2 (2.9)
limitations
Some people acted as though it was my fault	145 (97.3)	2 (1.3)	1 (0.7)
I have this illness

SSCI-8, Stigma Scale for Chronic Illness.

**Table 4:** Practical and emotional consequences (N = 146)

RADIX items	Frequency, *n* (%)		
	Strongly disagree/Disagree	Agree	Strongly agree
Practical Consequences
People treat me differently	17 (11.6)	52 (35.6)	77 (52.8)
I do not go out as much as I used to	22 (15.0)	48 (32.8)	76 (52.0)
I cannot do some of the things I used to do	42 (28.7)	62 (42.6)	42 (28.7)
I feel I have lost control over my life	28 (19.1)	62 (42.5)	56 (38.4)
Emotional Consequences
I get annoyed or frustrated with myself	54 (36.9)	45 (30.8)	47 (32.3)
I get very angry about what it is happening to me	48 (32.9)	53 (36.3)	45 (30.8)
I feel I have lost confidence in myself	53 (36.3)	53 (36.3)	40 (27.4)
I feel low or upset when I think about my condition	56 (38.4)	51 (34.9)	39 (26.7)
I find myself worrying about my condition	62 (42.4)	44 (30.2)	40 (27.4)

RADIX, Representations and Adjustment to Dementia Index.

### 3.1 Correlations between quality of life and other outcome measures

The QoL-AD score showed a positive correlation with GSES score and negative correlations with BDI-FS, RADIX emotional and practical consequences, SSCI-8, and BHS scores (Table 5).

**Table 5:** Spearman correlations between quality of life and other outcome measures

	QoL-AD rho (*p*)
MMSE	0.06 (0.46)
ADAS-Cog13	0.003 (0.96)
CDR-GS	0.13 (0.09)
Years of education	0.04 (0.60)
Disease duration	– 0.14 (0.07)
FAQ	– 0.04 (0.60)
BDI-FS	**– 0.45** (<0.0001)
BHS	**– 0.22** (0.006)
SSCI-8	**– 0.31** (<0.0001)
GSES	**0.34** (<0.0001)
SWLS	**0.44** (<0.0001)
Practical consequences RADIX	**– 0.38** (<0.0001)
Emotional consequences RADIX	**– 0.41** (<0.0001)

ADAS-Cog13, Alzheimer’s Disease Assessment Scale-Cognitive; BDI-FS, Beck Depression Inventory-Fast Screen; BHS, Beck Hopelessness Scale; CDR-GS, Clinical Dementia Rating-Global Score; FAQ, Functional Activities Questionnaire; GSES, General Self-Efficacy Scale; MMSE, Mini-Mental State Examination; QoL-AD, Quality of Life in Alzheimer’s Disease; RADIX, Representations and Adjustment to Dementia Index; SD, standard deviation; SSCI-8, Stigma Scale for Chronic Illness; SWLS, Satisfaction With Life Scale.

## DISCUSSION

Quality of life is a concept involving physical and mental health, cognitive performance, social interactions, and subjective well-being [23, 24]. Wehrman et al. [7] found that quality of life was the outcome most prioritized by patients with dementia or mild cognitive impairment and that this preference remained stable over time, regardless of the progression of cognitive impairment. Patients with AD can describe their beliefs and concerns throughout the disease trajectory [7, 8, 10, 25]. However, there is a lack of information regarding the subjective experiences of patients with early-stage AD [24].

In our study, depressive symptoms, feelings of hopelessness and stigma were frequent findings impacting on quality of life in a population with a short duration of illness (mean of 1.4 years since AD diagnosis) and minimal cognitive impairment (87.2% with a CDR-GS score of 0.5). Lima et al. [26] found that poor physical activity and functionality, depression, and anxiety were associated with low quality of life in a sample of 158 patients with mild AD. Patients with mild cognitive impairment also have a reduction in their quality of life in crucial domains including cognition, mood, social relationships, and helping with household chores [27]. Quality of life is already impacted in people with subjective cognitive decline, especially in the physical and mental health domains [28, 29].

Depressive symptoms are very frequent in patients with mild cognitive impairment with a prevalence between 16.9% and 55% and are associated with an increased risk of dementia [30– 32]. Depressive symptoms and memory impairment have traditionally been considered the main factors influencing the quality of life of patients with dementia [30, 31]. Negative attitudes about the future or hopelessness is conceived as the experience of anticipating unfavorable situations or consequences that are beyond a person’s control [33]. Hopelessness is recognized as a risk factor for self-harm and suicide [34]. In line with these results, 22% of patients in our study had depressive symptoms and 83% had moderate-to-severe hopelessness, implying a potential suicide risk.

We found almost 37% of patients had some stigma perception in our study. Stigmatizing attitudes have been associated with different neurological disorders contributing to low self-esteem, depression, and reduced health-seeking activity [35– 38]. People have beliefs about AD that are based upon their personal experience of the disease, as well as its depiction in the media and artistic works. Unfortunately, the latter often depict the most advanced cases with a high loss of functional independence. Different studies found that stigma is more pronounced in those with limited AD knowledge, those with little contact with AD patients, young people, and men [39]. On the other hand, we must also consider the internal stigma due to subjective beliefs and representations about the disease generated by patients themselves, especially in the early stages after diagnosis. Using the RADIX questionnaire [19] in our study, we could detect different emotional and practical consequences based on patients’ illness representations. Patients felt that people treated them differently and also reported a spectrum of negative feelings including anger, frustration, upset and worry.

Interestingly, the mean SWLS score in our study was 27.5, with 80.5% of patients considering themselves satisfied or extremely satisfied with life. Similar findings were previously reported in the literature [24, 27]. The decline in self-reported quality of life was not accompanied by the expected parallel decline in life satisfaction, for which a possible explanation could be that the QoL-AD and SWLS measure different constructs [24, 27].

Our study has some limitations. First, the perceptions of patients’ relatives or partners were not collected in this study. The discrepancy in the assessment of quality of life between self-report and proxy report has been described from the onset of cognitive impairment regardless of the instrument administered [40]. Therefore, we cannot rule out that the negative impact on quality of life has been underestimated in our study. Second, the cross-sectional design of the study does not allow us to identify any changes in the patient’s perception of quality of life over time [25]. Third, we acknowledge a potential selection bias given that those people most motivated to collaborate or with a better relationship with their physicians may have enrolled in the study. Finally, patients were recruited from memory clinics. It would be important to replicate these results in a population-based study.

The ability to live well with AD should be the main goal of multidisciplinary teams managing this disease, especially in the early stages where patients retain their awareness of the disease and can make key decisions for their future [41]. Different psychological resources have been described that could help patients deal with negative beliefs associated with the diagnosis of AD and uncertainty about the disease progression and loss of autonomy. Langer et al. [42] found that self-efficacy was associated with a better perception of quality of life in patients with mild cognitive impairment. In addition, resilient coping was associated with greater autonomy and better quality of life than non-resilient patients in a study in mild cognitive impairment [43]. Patients’ coping strategies and family cohesion and flexibility as well as good communication among their members moderated the relationship between psychological problems and quality of life [26].

Despite successive awareness campaigns and educational programs, lack of information and misconceptions about AD and mild cognitive impairment remain a critical problem [36– 39]. Therefore, as a strategy to reduce the impact of stigma on patients, more information campaigns are needed, especially targeting those population groups identified as having the largest knowledge gaps about AD [36].

### Conclusion

Stigma, depressive symptoms, and hopelessness are frequent scenarios in AD negatively impacting quality of life, even in a population with short disease duration and minimal cognitive impairment. Understanding patients' perceptions in the early stages of the disease may facilitate adopting specific strategies to develop psychological resources to foster living well with AD.
